# Moderated Online Social Therapy for Young People With Active Suicidal Ideation: Qualitative Study

**DOI:** 10.2196/24260

**Published:** 2021-04-05

**Authors:** Eleanor Bailey, Jo Robinson, Mario Alvarez-Jimenez, Maja Nedeljkovic, Lee Valentine, Sarah Bendall, Simon D'Alfonso, Tamsyn Gilbertson, Ben McKechnie, Simon Rice

**Affiliations:** 1 Orygen Parkville Australia; 2 Swinburne University of Technology Hawthorn Australia; 3 Centre for Youth Mental Health, University of Melbourne Parkville Australia; 4 School of Computing and Information Systems, University of Melbourne Parkville Australia

**Keywords:** suicide, youth, social media, internet-based intervention

## Abstract

**Background:**

Web-based interventions are a promising approach to support youth at risk of suicide, and those incorporating peer-to-peer social networking may have the added potential to target interpersonal states of perceived burdensomeness and thwarted belongingness. Owing to feasibility and safety concerns, including fear of contagion, this had not been tested until recently. In 2018, we conducted a pilot evaluation to test the feasibility, safety, and acceptability of a Moderated Online Social Therapy intervention, called Affinity, with a sample of young people with active suicidal ideation.

**Objective:**

The aim of this study is to report qualitative data collected from study participants regarding their experience of the web-based social network and the consequent safety features.

**Methods:**

Affinity is a closed website incorporating 3 key components: therapeutic content delivered via comics, peer-to-peer social networking, and moderation by peers and clinicians. Semistructured interviews were conducted with 17 young people who participated in the pilot study after 8 weeks of exposure to the intervention. Interview data from 2 young people who did not use Affinity were excluded from the analysis. The interviews were analyzed using thematic analysis, with the frequency of responses characterized using the consensual qualitative research method. The results are reported in accordance with the Consolidated Criteria for Reporting Qualitative Research checklist.

**Results:**

A total of 4 overarching themes were identified: a safe and supportive environment, the importance of mutual experiences, difficulty engaging and connecting, and the pros and cons of banning discussions about suicide. Interestingly, although Affinity was perceived to be safe and free of judgment, concerns about negative evaluation and triggering others were significant barriers to posting on the social network. Participants generally supported the banning of conversations about suicide, although for some this was perceived to reinforce stigma or was associated with frustration and distress.

**Conclusions:**

The results not only support the safety and potential therapeutic benefit of the social networking aspect of Affinity but also highlight several implementation challenges. There is a need to carefully balance the need for stringent safety and design features while ensuring that the potential for therapeutic benefit is maximized.

## Introduction

### Background

Globally, suicide is the second-leading cause of mortality in young people, defined as those aged between 15 and 24 years [1,2]. In Australia, it is the leading cause of death in youth, accounting for more than one-third of all deaths in this age group [3]. Suicidal ideation and behavior, including self-harm, are relatively common in young people, with approximately 10.6% and 1.2% of young people reporting past 12-month suicidal ideation and suicide attempts, respectively [4]. Suicidal ideation and behavior are risk factors for future suicidal behavior and suicide [5], along with mental disorders including, most significantly, a diagnosis of depression [6]. Moreover, interpersonal states, including perceived burdensomeness (feeling as though one is a burden on others) and thwarted belongingness (lacking a sense of belonging to any particular group) may also confer risk, as articulated by the Interpersonal Theory of Suicide [7,8]. Despite extensive investment and research into youth suicide prevention, the rates of suicide and suicide-related behavior in young people are rising both in Australia and internationally [3,9,10]. The reasons for this are complex, as such there is unlikely to be a single causal factor or solution. However, youth consultations suggest that there is a need to develop and evaluate new and innovative interventions that are theoretically based, target known risk factors for suicide, and are acceptable and relevant for young people [1,11].

The internet has increasingly received attention as a potential platform for the delivery of suicide prevention interventions for young people because of its reach, accessibility, acceptability, and cost-effectiveness [12-14]. Although several systematic reviews and meta-analyses have shown that digital interventions can reduce suicidal ideation and/or behavior in adults [15-18], far fewer studies have focused on developing and testing digital suicide prevention interventions for youth [12,19]. In addition, although the integration of digital interventions with existing mental health services may have several benefits, including improved treatment engagement and outcomes [20,21], to date there have been limited efforts to integrate web-based and face-to-face treatments for young people with suicidal thoughts or behaviors [22]. Finally, the internet has the capacity to facilitate mutual peer-to-peer support, which may in turn have the potential to target interpersonal risk factors for suicide (perceived burdensomeness and thwarted belongingness) [23]; however, no professionally developed intervention has included such a component. This may be in part because of concerns regarding the possible contagion of suicidal behavior, cyberbullying, and trolls [14,23].

### Objectives

To address these gaps while also mitigating the potential risks, researchers at Orygen in Melbourne, Australia, developed and pilot tested *Affinity*, a Moderated Online Social Therapy (MOST) [24-26] intervention designed as an adjunct to face-to-face treatment for young people with active suicidal ideation. Quantitative data, reported elsewhere [27], revealed Affinity to be safe, feasible, and acceptable, with exploratory correlations indicating associations between clinical improvement and key aspects of Affinity usage. Given the novel nature of the Affinity intervention, particularly its inclusion of a web-based social network, quantitative data alone are insufficient to understand important aspects of the user experience of the platform. Thus, the aim of this study is to qualitatively explore the views and experiences of young people who participated in the Affinity pilot study. Qualitative outcomes focused on the social networking aspect of the intervention, including moderation and safety features.

## Methods

### Design

This study reports qualitative interview data collected from young people who took part in a single-arm, pre-post test pilot study of the Affinity intervention conducted in 2018.

### Participants

Participants were current clients of a youth mental health clinic in Melbourne, Australia (the Youth Mood Clinic), that specializes in the treatment of young people with severe mood disorders [28]. The Youth Mood Clinic clients were referred to the study by their treating clinicians based on the clinician’s perception of their suitability. They were eligible to participate in the trial if they had experienced suicidal ideation within the past 4 weeks (screened for by the lead author using the question, “Have you had any thoughts of suicide in the past four weeks?”), had regular and ongoing internet and telephone access, and were able to give informed consent and comply with study procedures. Additional inclusion criteria to ensure participants’ safety and enable execution of safety protocols (if required) were as follows: well engaged with treatment and not approaching discharge in the next 4 weeks; familiar with, and willing to use, crisis supports; and willing and able to nominate 2 emergency contacts. There were no specific exclusion criteria related to the level of suicide risk, although clinicians were consulted on a case-by-case basis regarding participant suitability.

Of the 20 young people who participated in the pilot study, 17 (85%) completed a qualitative interview at follow-up. Of the 3 who did not complete a qualitative interview, 2 were not responsive to the research team’s contact attempts at follow-up (but remained engaged in their treatment at the clinical service) and 1 moved overseas before the qualitative interview could be completed. A total of 2 participants did not use the social network during the intervention period, engaging only with the therapeutic comic component of Affinity; therefore, their interview transcripts were excluded from the analysis.

The 15 remaining participants had a mean age of 21.3 years (SD 2.7; range 17-24), with 9 identifying as female, 5 as male, and 1 as transgender. At baseline, 93% (14/15) participants had suicidal ideation scores above the clinical cutoff on the Adult Suicidal Ideation Questionnaire [29] and 73% (11/15) reported at least one previous suicide attempt. On the Patient Health Questionnaire-9 [30], 80% (12/15) participants were in the *severe* or *moderately severe* range for depressive symptoms.

### Intervention

The Affinity intervention is described in detail elsewhere [23,27]. Affinity is a closed website that consists of 3 main components: (1) therapeutic content delivered in the form of illustrated comics; (2) peer-to-peer social networking; (3) moderation by clinical experts and young people with lived experience of mental ill health. Users create a profile using either their own first name or a pseudonym, with 67% (10/15) participants in this study choosing to use their real first name. Users can access Affinity 24 hours a day, as often or little as they like, and can use any or all of the components. Clinical and peer moderators promote engagement with the social network and therapeutic content and provide guidance, information, and emotional support to users. Although both clinical and peer moderators post publicly on Affinity and communicate with users via private message, only the clinical moderators are responsible for monitoring and managing clinical risk. Moderators checked Affinity for posts potentially indicative of clinical risk twice daily on weekdays and once daily on weekend days.

In addition to clinical moderation, the Affinity intervention and research design incorporated a number of safety features. First, posts containing keywords related to suicide risk (eg, suicide, suicidal, die) were automatically detected and blocked by the system, which would then trigger a notification to the lead author’s business mobile phone. Participants were made aware that this was accessed only during business hours. If notified, the lead author would immediately review the post in question and, if concerned about risk, respond according to the approved study safety protocol (see the following paragraph). Affinity also includes a vent post function, which allows users to select “I’m just venting” before making a post. When selected, the post appears masked by a warning to other users about the potentially inflammatory nature of the post, and users can then elect to view the post’s content. The vent post function is primarily designed to protect users from unwillingly viewing posts with swear words. Importantly, vent posts are not immune to the automatic detecting and blocking safety function; as such, posts expressing suicide risk could still be blocked even if “I’m just venting” is selected. Throughout the trial, 3 participants had at least one post that was automatically blocked. One of these posts communicated current suicidal ideation but not imminent risk, and the remainder of the blocked posts were not related to suicide risk but to other violent or aggressive behaviors that participants had experienced. Although the automatic blocking system used simple string matching and could not detect alternatives of keywords, such as sui*c*de, no participants in this study attempted to bypass the system.

A comprehensive safety protocol was in place outlining risk assessment and management procedures, including provisions for telephoning participants’ emergency contacts and/or emergency services, if risk was assessed to be high. All information about clinical risk identified during participation in the trial was communicated to the participants’ treating clinicians. Participants were also required to agree to a *terms of use* before being given access to Affinity, which included a request not to share personal or contact information with other users via the website to prevent conversations about suicide happening externally. These measures were in place to mitigate the risk of distress to participants or the contagion of suicidal behavior [23]. Accordingly, although participants were aware that the purpose of Affinity was to support young people who experience suicidal thoughts, they were actively discouraged from talking about suicide in the social network.

### Procedure

Participants were referred to the study by their treating clinicians between April and August 2018 and were given access to Affinity from the point of entry into the study until the intervention was closed (October 31, 2018). Semistructured interviews were conducted with each participant approximately 8 weeks after they were given access to Affinity by the lead author (EB), an experienced research assistant and a PhD candidate. EB had an established relationship with the participants in that she had also conducted the baseline assessments but was not involved in the delivery of the intervention itself (ie, did not post on Affinity or use it to communicate with users). The interviews were conducted either in a private room at the mental health service or in participants’ homes, with only the interviewer and interviewee present. The interview length ranged from 26 minutes to 75 minutes, with a mean duration of 48.3 (SD 16.4) minutes. Table 1 displays each participant’s study ID, age, interview length, severity of depression symptoms at baseline, and suicidal ideation score at baseline. To protect the identity of the transgender participant, participants’ genders are not provided. Participants were informed that the purpose of the interview was to explore positive, negative, and neutral feedback about Affinity, and were encouraged to be as open and honest as possible. As the purpose was to obtain feedback from as many participants as possible regarding their experience, data saturation was not assessed.

The interview schedule was designed to obtain participants’ views and experiences about Affinity, with prompts included if answers were vague. For the purpose of this paper, only responses related to the social network, safety features, and peer and clinical moderation were analyzed. The full interview schedule is provided in Multimedia Appendix 1. All interviews were audio recorded and transcribed verbatim, and potentially identifiable information was removed. Brief field notes were recorded during interviews.

This study was approved by the Melbourne Health Human Research Ethics Committee (ID 2017.187). All participants provided written informed consent. Participants under the age of 18 years were required to provide consent from their parents or guardians.

**Table 1:** Participant ID, age, interview length, and clinical characteristics at baseline (N=15).

ID	Age (years)	Interview length (min)	Baseline depression category (Patient Health Questionnaire-9)	Baseline suicidal ideation score (ASIQ)^a^
2	20	48	Severe	130
3	23	68	Moderate	42
5	22	50	Moderately severe	61
6	24	70	Moderately severe	24
7	23	71	Severe	130
8	23	32	Moderately severe	120
10	21	48	Severe	79
11	18	53	Moderately severe	119
12	24	46	Moderate	78
13	17	26	Severe	132
15	17	26	Moderately severe	69
16	17	34	Severe	136
17	24	75	Severe	78
18	23	39	Moderate	74
19	23	39	Severe	79

^a^ASIQ: The Adult Suicidal Ideation Questionnaire. The scale has a possible score range of 0-150; scores of 31 or more are considered to be in the clinical range.

### Data Reporting and Analysis

Data were reported in accordance with the Consolidated Criteria for Reporting Qualitative Research [31]. The checklist is provided in Multimedia Appendix 2. Only interview data pertaining to the social network, moderation, and safety features were analyzed for the purpose of this paper. Data were analyzed using inductive thematic analysis following 6 steps by Braun and Clarke [32]: (1) familiarizing with data, (2) generating an initial coding frame, (3) searching for themes, (4) reviewing themes, (5) defining and naming themes, and (6) reporting. The lead author (EB) read and reread the interview transcripts to immerse herself in the data, and based on this, an initial coding frame was generated. A coauthor (LV) checked the coding frame against a 10% subset of the data (ie, 2 interviews) and then discussed this with EB to refine the coding frame. The coding frame was then applied to the data provided by the lead author. Codes were then grouped into initial themes, which were reviewed and refined, with some combined and grouped into minor themes as necessary. SR, MN, and JR were regularly consulted regarding the codes and themes developed. The data were coded, and codes were grouped into themes by hand and then transferred to a web-based whiteboard platform (Miro) to produce hierarchical thematic maps, where they were organized, reviewed, and refined.

Data were reported according to the consensual qualitative research method [28], using the labels *few* (rare support; endorsed by 10%-20% of the respondents), *some* (variant support; endorsed by 21%-50% of the respondents), *many* (typical support; endorsed by 51%-90% of the respondents), or *most* (general support; endorsed by 91%-100% of the respondents).

## Results

### Overview

A total of 4 key themes were identified: (1) a safe and supportive environment, (2) the importance of mutual experiences, (3) difficulties in connecting and engaging, and (4) the pros and cons of banning discussions about suicide. These themes are outlined later and illustrated with exemplar quotes. Study ID and age are provided alongside quotes. The hierarchical thematic maps are available in Multimedia Appendix 3, and a table of themes, subthemes and/or codes, endorsement percentages, and exemplar quotes are available in Multimedia Appendix 4.

### Theme 1: A Safe and Supportive Environment

The first theme describes participants experiencing Affinity as a positive environment, wherein they felt both safe and supported. Indeed, many participants (11/15, 73%) specifically discussed feeling safe on Affinity. This sense of safety was discussed both in terms of being shielded from negative content and feeling safe from ridicule or judgment. A few participants (2/15, 13%) also referred to feeling safe because they knew their privacy was protected.

Some participants (6/15, 40%) specifically referred to the presence of clinical moderators as contributing to the sense of safety, in that users knew “if something did happen that there were people there that could step in” (ID 08, aged 22 years). A few participants (3/15, 20%) also reported feeling safe because they knew that all users were well-intentioned, with one stating they knew other users “weren’t there to harm me or affect me negatively in any way. They were just there to better themselves*”* (ID 02, aged 20 years). This belief in the good intentions of other users was partly attributed to their mutual experiences, although this was identified as a distinct theme and is therefore elaborated separately later.

Some (6/15, 40%) specifically labeled Affinity as a *friendly* and/or *supportive* space. Many (8/15, 53%) participants said that they had provided support to other people on Affinity; 6 of these said this was a positive experience, with 1 participant noting “…it feels good to know you've helped someone” (ID 05, aged 22 years). Two of these participants noted negative impacts in addition to discussing positive outcomes of supporting others, with one stating “it can be frustrating when people don't want to listen to what you've got to say” (ID 07, aged 23 years), and another referring to their tendency to worry about other people at the expense of “fully looking after myself” (ID 17, aged 23 years).

Participants also expressed appreciation for the way the peer and clinical moderators connected with them and the social network. Many (8/15, 53%) valued when moderators reached out with personalized messages to check on them or suggest particular therapeutic components to try within Affinity:

[The moderators] sent us really big paragraphs which I really liked. I don't care how long they are, but the fact that they took time to write that and it was meaningful, it was really supportive.

ID 16, aged 16 years

Although many participants (9/15, 60%) also appreciated the effort the moderators put into facilitating group conversations and welcoming new users, some participants (4/15, 27%) felt they were overly enthusiastic in their attempts to promote connections within the network:

They were good. For one thing, it made it so that there was no post that was really ignored...But I will say that there were points where it felt a bit artificial to an extent.

ID 11, aged 18 years

Some participants (6/15, 40%) specifically drew comparisons between Affinity and other mainstream social networking sites, such as Facebook, stating that Affinity felt safer and more supportive:

People feel more secure being open in that area, as opposed to other social media. They could be attacked or feel triggered. But in Affinity, we're all here to support each other.

ID 03, aged 22 years

When I'm in a good mood Facebook was great. But when I'm in a bad mood or have anxiety, I'm not sure, something about it upsets me. I never got that from Affinity, I'm not sure if it’s because I didn't know the people, or it’s because the posts were positive, or because I knew they were going through the same thing I was going through. Whatever it was, I never felt upset.

ID 12, aged 23 years

For some (7/15, 47%), Affinity was therefore perceived to provide a way for safely and easily interacting, or connecting, with others, which was particularly helpful in periods of low mood or isolation when any form of interaction was difficult:

I mean, for me I feel very disconnected from the world, so I feel a little bit more connected when I'm on Affinity. Just a little bit more. It's not fixing anything per se but I do feel a little better using it.

ID 06, aged 24 years

Previous, what I would normally do is just cut contact off completely. This gave me one thing that I could stay on.

ID 12, aged 23 years

### Theme 2: The Importance of Mutual Experiences

Many participants (13/15, 87%) specifically spoke about valuing participation in a network of other people with similar lived experiences (namely, depression and suicidal thoughts). Many (8/15, 53%) specifically spoke about how this helped them to feel less alone or *crazy* with regard to these experiences:

You read posts and it would be something, you'd be like oh I've had that thought or that's how I feel. Then in your head you're like oh, this person's feeling that way too. [...] You read that post and think oh okay I'm not crazy, I'm not the only person that thinks that.

ID 08, aged 22 years

Talking to other people that actually feel what I do is - I mean yeah, it's pretty good. I think the most terrible thing about depression is it makes me feel like I'm the only person in the world who feels that and it makes me feel lonely.

ID 10, aged 21 years

Some (7/15, 47%) also discussed how this led to them feeling particularly validated and understood by the other users, with one stating:

That was something I could talk about on Affinity, and be candid about on Affinity and not - I could tell my friends, but there was - it was - there was something about saying it in front of other people who understood about this.

ID 11, aged 18 years

Some participants (4/15, 27%) also reported that they were able to learn from other users, because they either provided relevant advice or a new perspective or posted about how they had dealt with their own problems. For example, one participant reflected that suggestions from other users were “more useful than people that haven't been through this kind of thing, purely because they themselves know more or less what helps and what doesn't” (ID 05, aged 22 years). Another user discussed how responses to a different user’s post about a “problem that was similar to mine...helped me see things from the other person’s side of view and look at their problem in a different way” (ID 02, aged 20 years). A few participants (3/15, 20%) said that hearing about the experiences of others was encouraging in terms of their own ability to “get through this.” One participant said that knowing the peer moderators had lived experience of mental ill health and that they were in recovery “gives a little bit of hope*”* (ID 02, aged 20 years).

Finally, some participants (4/15, 27%) specifically stated that they experienced many of the benefits related to mutual experiences even though they did not actively engage in social networking; in other words, just reading posts was beneficial. One participant said that even though they did not post, “it helped to just read someone else's post about how they were feeling” (ID 17, aged 23 years).

### Theme 3: Difficulties in Engaging and Connecting

Participants also reported that they encountered barriers to engaging with the affinity social network and/or connecting with other users. These barriers have been categorized into subthemes of internal barriers (related to feelings of anxiety or apprehension) and external barriers (attributes specific to Affinity).

#### Internal Barriers to Engaging and Connecting

Many participants (12/15, 80%) discussed feelings of anxiety or apprehension related to posting on the network and/or replying to posts made by others. A total of 2 subthemes related to the source of this anxiety were identified: fear of negative evaluation and fear of causing harm.

##### Fear of Negative Evaluation

Many (8/15, 53%) participants discussed feelings of anxiety related to posting or replying to posts on Affinity, often related to concerns that other users would not care about their post or that no one would respond to their disclosure or comment. For example, one participant (ID 18, aged 23 years) discussed not wanting to be “the first one to comment,” for fear that other users “won’t react to it and you'd just be sitting there and no-one will give a shit about what you said.” Some participants (4/15, 27%) said this uncertainty manifested as “overthinking,” with one participant stating, “I just kept second guessing whatever I would write whenever I wanted to make a post” (ID 02, aged 20 years).

One participant (ID 17, aged 23 years) likened this to anxiety associated with posting on other forms of social media, stating “social media has ruined people, and they're like, ah, is this going to get likes, or are people going to comment back to this?” Indeed, some (5/15, 33%) specifically stated that these thoughts and feelings were typical for them and extended to all social media and did not attribute them to Affinity specifically.

Some participants (4/15, 27%) expressed confusion about the fact that they felt anxious despite the supportive nature of the social network, with one saying*,* “It’s weird because we know that it’s a safe place, and yet we still get very anxious about those things” (ID 19, aged 23 years). Another said, “I don’t know why I was being super scared, because I have all this proof that everybody on there is actually really supportive and nice and nothing that I’m posting is too bad or controversial” (ID 02, aged 20 years).

##### Fear of Causing Harm

Some participants (6/15, 40%) reported that they held back from posting or replying to posts because they were worried about causing distress or being unhelpful to others, with 2 users expressing uncertainty regarding permitted posts:

I guess I didn't even post things, like thoughts that I might be having, no one likes me or I'm just a complete failure or life's not worth living, I feel so hopeless, I feel like I don't have a future. I wouldn't say stuff like that, because I thought it would be too triggering for other people.

ID 07, aged 23 years

I felt even if I had something to say, I didn't feel comfortable saying it. I wasn’t sure if I wrote something if it’d make it worse, or I'm not sure how to feel about giving other people advice. So, I kind of deliberately took a - even if I thought I had advice, I wouldn't give it, because I'm not sure how the reaction would be.

ID 12, aged 23 years

One participant suggested that this uncertainty could have been better managed if Affinity had contained a list of topics or phrases that were permitted and not permitted or if moderators had initiated group discussions around sensitive issues.

#### External Barriers to Engaging and Connecting

In total, 2 external barriers were identified: the small user base and the impersonal nature of the interactions with other users.

##### Small, Inactive User Base

Many participants (9/15, 60%) stated that there were too few users on Affinity and/or that the user base was too inactive. A few participants (2/15, 13%) specifically said that this reduced their motivation to log in altogether because “there isn’t much happening” (ID 06, aged 24 years). A few (3/15, 20%) stated this hindered willingness to post because they anticipated a delayed response, and 2 participants reported that a lack of timely or prompt acknowledgment or validation from the network in relation to comments or disclosures made on Affinity had a negative impact:

You can post something and go unnoticed for two days. So, in that aspect, it [...] felt almost a little counterproductive [...]it just didn't necessarily help with the isolation.

ID 05, aged 22 years

Just say I type something, and no one responded within a day, I'm like, oh, they don't care about me. Oh, they don't want to listen. I made them feel shit, or something. Those thoughts keep going around in my head, and I'm like, oh, I shouldn't have said that.

ID 16, aged 16 years

##### Impersonal Interactions

Some participants (7/15, 47%) expressed dissatisfaction with their inability to interact more closely or on a long-term basis with other users. Specifically, the prohibition of sharing personal or contact information, the lack of a private user-to-user chat function, and the temporary nature of Affinity were perceived to have contributed to difficulties establishing meaningful connections with other users:

It probably helped a little bit and made me feel connected and heard, but not like - it didn't, you know, completely erase my feeling of isolation and loneliness because at the end of the day, it's a temporary thing, it's not permanent, I don't know who anyone is on there.

ID 07, aged 23 years

### Theme 4: Pros and Cons of Banning Discussions About Suicide

The fourth general theme was related to the fact that both positive and negative feedback was received regarding the banning of suicide-related posts on Affinity. A total of 11 participants specifically discussed this policy; of these, 7 (64%) generally supported it, 2 (18%) did not, and 2 (18%) mentioned both positive and negative impacts. Participants in support of prohibiting suicide-related posts stated that they felt conversations about suicide could be harmful or *triggering* for themselves or other users, with one stating:

For me, it would be very triggering if I were to see anything about suicidal thoughts. But that depends on the person I guess because I think I'm just very emotional, and very easily influenced.

ID 19, aged 23 years

Some participants (3/11, 27%) specifically stated that they valued the *vent post* function of Affinity.

One participant with mixed views about these terms of use stated:

It’s definitely a little bit weird that [Affinity] is for people who are feeling suicidal but you can’t really talk about it or - definitely seems like it had sort of a stigma as well. But I feel like it was good that you didn’t let us talk about it like that because I feel like if I saw somebody talking about suicide, then it would get me thinking. Or I’d be super worried about them and I don’t think either of those are helpful for me or anybody else on the website.

ID 02, aged 20 years

Another participant suggested that banning discussions about suicide could be challenging for people who *need to talk about* their suicidality. Indeed, some participants (3/11, 27%) spoke about needing to vent without necessarily needing a crisis intervention, for example, one said:

I know for myself sometimes when you want to - you just need to say something and to some people it's going to sound really bad but you're genuinely like I just need to get this out of my head.

ID 08, aged 22 years

One participant said it would have been helpful for them if they had been able to engage in discussions openly about suicide on Affinity:

So when I was alone and I was able to be on my phone and look through, it just would have helped [...] to have other people share their actual experiences.

ID 17, aged 23 years

A few participants (2/11, 18%) described the experience of having a post automatically blocked by the system and both found it a negative experience, mostly because of confusion about why it was blocked in the first place:

It was just like annoying. I was just like, really? Especially [because] there was no real obvious reason as to why it got blocked. I feel like for me if it was an obvious reason I'd be like okay, I can change that, but I'm looking at it going I can't actually change anything in this to make it any less - different to what it is.

ID 08, aged 22 years

I thought somebody took time out of their life to block my - or report me. I was like, are you serious? [...] Yeah, just seems like nothing now, but at the time I got really upset. Other people were using swearing and stuff. How can F-U-C-K be involved but not death? That's what I didn't understand, but yeah, I get it now.

ID 16, aged 16 years

## Discussion

### Principal Findings

This study used thematic analysis to examine the experiences of 15 young people who used the therapeutic web-based social networking platform, Affinity. We found that participants experienced Affinity as a safe and supportive environment where they felt less alone and understood by others, yet also experienced barriers to fully engaging and connecting. We also found that although participants generally supported banning discussions about suicide, some potential adverse effects were noted.

### Positive Appraisals of the Affinity Social Network

The finding that Affinity was perceived to be supportive and safe and that participants valued being surrounded by others with similar experiences aligns with the quantitative results we have reported elsewhere supporting the acceptability and safety of Affinity [27]. The importance of mutual experiences has also been identified as a theme in other studies using the MOST platform [33,34] and the literature on online support groups for suicidal people [35,36]. The positive impact of providing support to others was also raised by some participants and has been identified in the broader online support group literature [36], although it is acknowledged that 2 participants in this study also reported negative aspects of supporting others, which are important and worthy of further exploration. For example, phenomena such as compassion fatigue or burnout may impact young people supporting other young people on Affinity; this has been hypothesized to occur in trained volunteers from at least one anonymous online forum for suicidal people [37].

Previously, we theorized that the peer-to-peer support facilitated by Affinity could mitigate the key risk factors described in the Interpersonal Theory of Suicide [7,8] of thwarted belongingness (via immersion in a network of similar others) and perceived burdensomeness (by enabling users to help others in need) [23]. The feeling of being less alone reported by participants suggests that they may have experienced an increased sense of belongingness; this aligns with our quantitative results showing a significant and large effect size (Cohen *d*=−0.96; *P*=.006) improvement in thwarted belongingness [27]. Regarding the possible influence of Affinity on perceived burdensomeness, as noted earlier, several participants reported that providing support to others was a positive experience. Although limited data preclude a deeper exploration of this, it is possible that helping others may effectively lessen the sense of burdensomeness [23]. Conversely, however, it may also be that fear of causing harm to others, either by triggering them or by providing advice perceived to be *bad* in response to a post, may also serve to increase burdensomeness. Therefore, further studies are warranted. Overall, these findings attest to the significance of the social networking component of Affinity and that it may have therapeutic benefits in and of itself. Moreover, some participants reported experiencing these benefits just by reading other users’ posts, suggesting that the positive impact of mutual experiences may occur even without active participation. The ability of participants to experience therapeutic benefit from passively participating in web-based peer support networks has also been found in previous studies using the MOST model [33,38] and in studies of online health-related support groups more broadly [39,40].

### Barriers to Engaging and Connecting

Despite positive appraisals, participants also reflected on experiencing barriers to engaging and connecting in the trial. Perhaps unsurprisingly, given that most participants had depression symptoms in the severe or moderately severe range, fear of negative evaluation was a key barrier to engaging with the social network. Research has shown a link between symptoms of depression and a tendency to participate passively (or *lurk*) in web-based social networks [41-44]; indeed, participants in this study likened their apprehension about posting or commenting on Affinity to how they feel about social media more generally. Despite Affinity’s vent post feature, which 3 participants specifically discussed in positive terms, a key barrier to engaging with the social network was fear of causing harm or triggering others. Participants also reported an inability to properly connect with other users, attributed to the finite period of access to Affinity as well as the lack of one-to-one chat and ban on sharing contact details. Building presence in a social network can be a significant investment of an individual’s effort and time, and this was likely negated in this study, given the short intervention period and lack of opportunity to connect with users beyond the intervention. Although these safety and design choices were deliberately made because of the novel nature of the intervention and the high-risk nature of the sample, these will likely be carefully relaxed in future iterations of Affinity.

### Balancing Risks and Benefits of Talking About Suicide

The finding that many participants supported the prohibition of suicide-related posts is somewhat surprising; we expected participants may have experienced this feature as authoritarian based on research suggesting that young people want to talk about suicide with one another and with adults [45]. This previous research, however, was conducted with young people who were not currently at risk. It is possible that young people with active suicidal ideation are more likely than those who have recovered to find open discussions about suicide distressing, particularly where suicidal cognitions are experienced as intrusive and involuntary. Indeed, concerns about becoming distressed or suicidal themselves were cited as key reasons for supporting the prohibition of discussions about suicide. Despite this, several adverse effects associated with banning discussions about suicide have also been raised by the participants. For example, one participant suggested that this may serve to perpetuate stigma. Given that openly talking about suicide to alleviate stigma and encourage help seeking is the rationale underpinning suicide prevention media campaigns [46], the potentially stigmatizing nature of prohibiting conversations about suicide on Affinity is worthy of consideration. Participants also suggested that talking about suicidal thoughts may be helpful for some people, and one participant said it would have been helpful to see how other people on Affinity deal with their suicidal thoughts; possibly, the benefits associated with mutual experiences would be heightened if these discussions were permitted. In addition, the participants who experienced having a post automatically blocked by the system reflected that this was a negative and confusing experience for them. This way, the automatic blocking system used in the Affinity pilot may actually be counterproductive and lead to increased distress, particularly for users who are already in a distressed state when posting. In the pilot, the list of risk words that would trigger a post to be blocked was not disclosed to participants nor were they informed which words had triggered the blocking after the fact. It is therefore possible that communicating this information to participants would have reduced their distress and frustration in response to having a post blocked. Given the mixed responses to the banning of discussions about suicide, more research is required to determine under what circumstances, by what mechanisms, and for whom, talking about suicide on platforms such as Affinity may be helpful or harmful. One possibility is that young people actively trying to suppress or avoid their suicidal thoughts as a coping strategy might experience anxiety or distress when encouraged to discuss them [47].

### Implementation Challenges and Future Directions

Although the first 2 themes are indeed encouraging and provide further support for the acceptability, safety, and potential therapeutic benefit of Affinity, the barriers to engaging and connecting and mixed views on banning discussions about suicide pose a number of implementation challenges. First, there is a question of how to promote active participation in the social network to a population of users who may be particularly sensitive to judgment from others and who may perceive social media as being *unsafe*. Indeed, previous research has identified that adolescents tend to view social media as judgmental and threatening, particularly where they may be affected by mental illness, making them more susceptible to these cognitions [48,49]. Other challenges relate to how to allow users to connect more personally with each other and how to allow discussions about suicide to occur while maintaining adequate safety standards. There is clearly a careful balance to be struck so that the potential benefits of Affinity, particularly those related to mutual experiences, can be maximized while still protecting users who may be more susceptible to feeling distressed or triggered. This tension, associated with balancing safety features and ensuring potential benefits are maximized, is common to internet-based suicide prevention intervention research more broadly [50]. Given the quantitative [27] and qualitative findings of this study supporting the safety of Affinity, there is an opportunity to carefully relax some of the safety features used in the pilot study in the future. The inclusion criterion related to participants being sufficiently engaged with the clinical service, which in this study was implemented to ensure safety protocols could be appropriately executed, could potentially be relaxed in future iterations so that young people who are less well engaged in treatment could provide access to this potentially helpful intervention. Other options for consideration in future iterations of Affinity could include scheduled, moderator-facilitated discussions about suicidal thoughts focusing on helpful strategies and stories of hope and the promotion of guidelines about how to safely talk about suicide [51], including specific advice about unacceptable content (eg, threatening suicide, inciting suicide in others). Importantly, users should be able to avoid discussions about suicide altogether on Affinity, without fear of judgment. In the absence of evidence-based guidelines for implementing digital interventions for this population, decisions regarding these features should be theoretically and empirically driven, consumer led, and carefully evaluated in an ongoing way regarding their acceptability and safety.

### Limitations

Several limitations should be considered when interpreting the findings of this study. First, the interviews focused on breadth, rather than depth of data; as such, we were unable to explore in great detail the themes and subthemes that were identified. Future research is warranted to explore some ideas in detail; for example, understanding moderators of engagement and different engagement profiles would be important for the development of targeted consumer-informed web-based interventions for this population. Second, the sample size of 15, which is typical for qualitative research, prevents generalization of these findings beyond this study. Third, it is acknowledged that qualitative methods bring a degree of researcher subjectivity, which may have influenced the analysis and interpretation of results. To address this, regular discussions between members of the research team were held throughout the analysis process. Fourth, participants were not asked to review the transcripts or the study findings; however, as the transcripts were recorded and transcribed verbatim, the likelihood of error was minimal. Moreover, given the difficulty associated with contacting participants in this study, it was not appropriate or feasible to request that they provide feedback on the findings. Fifth, we did not analyze relationships between the themes, for example, whether participants who thought they benefited from passive participation were also those who experienced fear of negative evaluation in relation to posting; this should be a priority for future research. Finally, we did not include a measure of social anxiety, which would have shed more light on participants’ experience of barriers to posting; this should be a focus of future evaluations of Affinity.

### Conclusions

This study provides important preliminary data on the user experience of a web-based intervention for young people at risk of suicide, incorporating a social networking component and strict safety features. The findings suggest that the social networking component of Affinity is both safe and may possibly have therapeutic benefit, although participants experienced barriers to engaging and connecting related to their underlying feelings of anxiety as well as to design and the safety features of Affinity. Therefore, there is a need to carefully balance ensuring participants’ safety with maximizing the potential for therapeutic benefit. That participants experienced benefit despite the ban on conversations about suicide and that passive participation was reported to be beneficial suggest that just knowing one part of a network of similar others may be in and of itself therapeutic for young people at risk of suicide. Future iterations of Affinity should consider carefully relaxing the safety features while continually monitoring and evaluating their acceptability and safety; these decisions should be based on the theoretical and empirical literature and made collaboratively with consumers.

## Appendix

Multimedia Appendix 1 Interview schedule.

Multimedia Appendix 2 Consolidated criteria for reporting qualitative research checklist.

Multimedia Appendix 3 Hierarchical thematic maps.

Multimedia Appendix 4 Summary table of themes, codes, percentage endorsed, and example quotes.

## Abbreviations

MOST: Moderated Online Social Therapy
