# Correction: Moderated Online Social Therapy for Young People With Active Suicidal Ideation: Qualitative Study

**DOI:** 10.2196/29645

**Published:** 2021-06-10

**Authors:** Eleanor Bailey, Jo Robinson, Mario Alvarez-Jimenez, Maja Nedeljkovic, Lee Valentine, Sarah Bendall, Simon D'Alfonso, Tamsyn Gilbertson, Ben McKechnie, Simon Rice

**Affiliations:** 1 Orygen Parkville Australia; 2 Swinburne University of Technology Hawthorn Australia; 3 Centre for Youth Mental Health, University of Melbourne Parkville Australia; 4 School of Computing and Information Systems, University of Melbourne Parkville Australia

In “Moderated Online Social Therapy for Young People With Active Suicidal Ideation: Qualitative Study” (J Med Internet Res 2021;23(4):e24260), the authors noted one error.

Incorrect qualifications were provided for author Ben McKechnie. The original qualifications provided were: BA, MPsych(Clin), MClinFamTher. However, MClinFamTher was provided in error and has been removed.

The correction will appear in the online version of the paper on the JMIR website on June 10, 2021, together with the publication of this correction notice. Because this was made after submission to PubMed, PubMed Central, and other full-text repositories, the corrected article has also been resubmitted to those repositories.

