# Supplementary material for: Moderated Online Social Therapy for Young People With Active Suicidal Ideation: Qualitative Study
Source: J Med Internet Res. 2021 Apr 5;23(4):e24260. doi: 10.2196/24260 (PMC8056298; doi:10.2196/24260)
Supplement: Multimedia Appendix 1 [file jmir_v23i4e24260_app1.docx]

**Multimedia Appendix 1: Interview Schedule**

| **Question** | **Prompts** |
| --- | --- |
| **Meaning and benefits of Affinity** | |
| How did you get involved in Affinity? | What made you interested in signing up? What were you hoping to get out of being involved? |
| Now that you’ve had a bit of time to use Affinity, how do you feel about it? | What was it like to be on Affinity?  What is Affinity to you?  What role does it have in your life?  How did you make Affinity work for you? |
| Why did/didn’t you use Affinity? | [Follow up with another 4 whys to understand the underlying motivations/drivers for use.] |
| **Usage of Affinity** | |
| Do you remember a specific time that was meaningful to you when you were using Affinity? | Can you tell me about it? |
| Do you remember a time that Affinity helped you solve a problem, question, or issue you were having? | Can you tell me about it? |
| Think back to when you used Affinity the least. What was going on for you at this time? What prompted you to go on to Affinity? | [If prompted by a moderator therapy suggestion or peer worker message] Was this helpful? What did you get out of going on to Affinity at this time?  [If prompted by an internal trigger, e.g., emotional distress]. Did you get what you were looking for on Affinity? [Prompt if no: What could we have done to help you get more out of Affinity at this time?] |
| Think back to when you used Affinity the most. What was going on for you at this time? What prompted you to go on to Affinity? | What kept you from using Affinity? Is there anything we could’ve done to help you get more out of Affinity during this time? |
| **Components of Affinity** | |
| What did you like most about Affinity? | Why? |
| What didn’t you like about Affinity? | Why? |
| Did you feel Affinity was relevant to you and your needs? | Was there enough suicide-related content?  What could we change to make it more relevant to you? |
| ***Social networking*** | |
| What did you think of the social networking aspect of Affinity (e.g. Café).? | If not used - What could we have changed to make you want to use it more? |
| What was it like being able to interact with other young people on Affinity? | What was it like interacting with other young people who had *experienced suicidal thoughts*?  Do you remember a time that you spoke with another person on the site that was meaningful to you?  Did you feel you were able to talk about the things that were important to you?  Did you provide support to other people? What was it like?  Did you receive support from others? What was it like? |
| Did you make meaningful connections? | [If yes] Can you tell me about this? How did you create these connections? What made the connection meaningful to you?  [If no] What would have made it easier to make meaningful connections? Why? |
| What was it like to have moderators on Affinity? | Peer moderators?  Clinical moderators?  What was helpful/unhelpful about the moderation?  Did the moderators influence your use of Affinity?  Did the moderators influence your interaction with other users on Affinity? |
| ***Therapeutic content*** | |
| What did you think of the therapeutic comics (“steps”)? | [If done] How were they helpful/relevant for you? [OR] Why weren’t they helpful/relevant for you? What could we change to make them more helpful/relevant/easier to do?  [If not done] What could we have changed to make these more interesting for you? |
| What did you think of the Do its? | [If done] How were they helpful/relevant for you? [OR] Why weren’t they helpful/relevant for you? What could we change to make them more helpful/relevant/easier to do?  [If not done] What could we have changed to make these more interesting for you? |
| What did you think of the Strengths part of Affinity? Looking for them, and levelling up? | [If done] What was it like for you to identify and/or level up your strengths on Affinity? What was helpful about it? What wasn’t helpful? Why/why not? What could we have changed about it?  [If not done] Did you see the find your strengths activity on Affinity? What did you think of it? What could we have changed to make this more interesting for you? |
| **Safety and Support on Affinity** | |
| Did you feel safe on Affinity? | Tell me about this – why did you give that answer? |
| Did using Affinity ever lead to you feeling distressed/upset? | Can you tell me about this? What happened? What did others do? How did this impact you/your use of Affinity? |
| *[If the participant ever had a post blocked by the system]:* What did you think about this post/s being blocked? | How did you feel about it? |
| Did you have any other negative experiences using Affinity? |  |
| **Ideal use of Affinity** | |
| If Affinity worked like Magic, with no limits on what it could do, what would it look like? | Use prompts to understand why the young person wants particular features, and how they would use this dream Affinity |
| **Affinity in the context of other mental health care** | |
| Can you tell me a little about your experience of mental health care over the last year? | Where does Affinity fit in with that?  How was it similar/different from other services you’ve used? |
| What do you think it would have been like to have been able to use Affinity in-session with your clinician at Orygen? | Tell me more about that |
| [If clinician not on Affinity] Would you have liked to have had your clinician at Orygen on Affinity?  [If clinician on Affinity] What was it like to have your clinician at Orygen on Affinity? | Tell me more about that |
| If Affinity and face-to-face support worked together like magic, what would this be like? | [Additional clarification probes if needed only]: What sorts of things would it allow you and your clinician to do? For example, would you have liked your clinician to be able to suggest therapy comics to you or communicate with you between sessions using something like Affinity?  [Ask additional probes to understand why the young person would want Affinity and face-to-face support to work a particular way, and how they would use this dream Affinity]. |
| **Closing Questions** | |
| Do you have anything else to add about your experience? |  |
