# Supplementary material for: Moderated Online Social Therapy for Young People With Active Suicidal Ideation: Qualitative Study
Source: J Med Internet Res. 2021 Apr 5;23(4):e24260. doi: 10.2196/24260 (PMC8056298; doi:10.2196/24260)
Supplement: Multimedia Appendix 3 [file jmir_v23i4e24260_app3.docx]

**Multimedia Appendix 3: Hierarchical thematic maps**

**Theme 1:**

**Theme 2:**

**Theme 3:**

**Theme 4:**
