# Supplementary material for: Moderated Online Social Therapy for Young People With Active Suicidal Ideation: Qualitative Study
Source: J Med Internet Res. 2021 Apr 5;23(4):e24260. doi: 10.2196/24260 (PMC8056298; doi:10.2196/24260)
Supplement: Multimedia Appendix 4 [file jmir_v23i4e24260_app4.docx]

**Multimedia Appendix 4: Summary table of themes, codes, percentage endorsed, and example quotes**

| **Theme** | **Sub-themes/codes** | **Example quotes** |
| --- | --- | --- |
| Safe and supportive place | Safe (73%) from harm and judgment.   - Moderators (40%) and users (20%) contribute to sense of safety - Knowledge that information is safe (13%) | “I think it was just that knowledge that it was a safe space”  “I also felt safe because there were the moderators” |
|  | Friendly and supportive (40%)   - Good to support others (40%), but can also be negative (13%) - Valued moderators sending personalised messages or suggest content (53%) - Effort of moderators appreciated (60%), but sometimes too much (27%) | “It had a good atmosphere, like the people on there were very friendly”  “Most of the time [supporting others] felt kind of nice. It can also be frustrating when people don't want to listen”  “There were points where [the moderators] felt a bit artificial” |
|  | Better than other social networking sites (40%) | “It just feels more secure than being on other different types of media, like Instagram or Snapchat” |
|  | An easy way to connect or interact (47%) | “It does give me a little bit of a feeling of connection, especially if I'm cooped up all day inside” |
| The importance of mutual experiences | Less alone/crazy (53%) | “It's just about not feeling so alone in it. Not being so isolated in at all.” |
|  | Validated and understood (47%) | “knowing that there's people that can understand” |
|  | Can learn from others (27%) | “I found it very useful to be able to hear the way that other people were dealing with…” |
|  | Sense of hope (20%) | “you feel like okay, I can get through this” |
|  | Benefits even with passive use (27%) | “Having somewhere where you sort of socially interacted, even if I didn't post anything...” |
| Difficulties engaging and connecting | Internal barriers (anxiety or apprehension) (80%)   - Fear of negative evaluation (common in other social media platforms, confused why they felt like this) (53%) - Fear of causing harm (40%) | “I know that those people would never judge me because stuff that they were posting, they were lovely. But I just have this really big fear of being judged.”  “I just didn't want to trigger others, so I refrained from talking a lot about what I was really feeling.” |
|  | External barriers   - Small and inactive user base (reduced motivation to log in and willingness to post, lack of timely response negative impact for some) (60%) - Impersonal interactions (lack of private chat, inability to share contact details, time-limited access period) (47%) | “There needs to be a bigger amount of people using it.”  “I'd like to be able to directly talk to someone”  “I couldn't fully socially connect with someone because I couldn't just be like […] here's my number” |
| Pros and cons of prohibiting suicide posts | There are pros (60%), including concerns about being triggered or unable to help.   - Vent post valued (20%) | “It would be very triggering if I were to say see anything about suicidal thoughts” |
|  | There are cons (27%), including perpetuation of stigma, the need to vent, wanting to hear other’s experiences.   - Having a post blocked was a negative experience (13%) | “Would have helped just to have other people share their actual experiences”  “I thought somebody took time out of their life to block my - or report me. I was like, are you serious?” |
